# Habitat Quality, Conservation Gaps, and Environmental Suitability of Siberian Cranes (*Leucogeranus leucogeranus*) in the Erlongtao River Basin, Songnen Plain

**DOI:** 10.3390/biology15191759

**Published:** 2026-10-02

**Authors:** Yang Wen, Haibo Jiang, Shiying Zhu, Junqi You, Huiyun Yue, Hanbo Teng, Chunguang He

**Affiliations:** 1 Key Laboratory of Environmental Materials and Pollution Control, the Education Department of Jilin Province, College of Engineering, Jilin Normal University, Siping 136000, China; 2 Key Laboratory of Wetland Ecology and Vegetation Restoration, Ministry of Ecology and Environment, Northeast Normal University, Changchun 130117, China; 3 Key Laboratory for Vegetation Ecology, Ministry of Education, Northeast Normal University, Changchun 130117, China

**Keywords:** land use change, MaxEnt, InVEST, wetland restoration, migration stopover sites

## Abstract

Wetland degradation induced by climate change and human disturbance poses severe threats to migratory waterbirds, particularly the critically endangered Siberian crane (*Leucogeranus leucogeranus*). The Erlongtao River Basin, a key stopover wetland complex on the Songnen Plain, exhibits prominent mismatches between general waterbird habitat quality and species-specific habitat suitability, together with widespread conservation gaps. In this study, line-transect and point-sampling field surveys were conducted during spring and autumn migration (2020–2024). The eight phases of land use datasets (1990–2023), the InVEST Habitat Quality module, and the MaxEnt habitat suitability model were used to quantify 33-year spatiotemporal dynamics of habitat quality and contributions from land use transitions, map spatial patterns of Siberian crane suitable habitats, and identify conservation gaps within and outside two national nature reserves. Wetland shrinkage and swamp fragmentation degraded regional foraging and habitat conditions for waterbirds. Transitions from unused and construction land to water bodies, shallows, and swamps increased habitat quality, whereas cropland expansion and soil salinization drove habitat degradation. In 2020, high-, moderate, and low-suitability habitats for Siberian cranes occupied merely 0.18%, 1.37%, and 3.55% of the total watershed area, respectively. Dry farmland constituted 47.97% of high-suitability habitats, 44% of which belonged to the poor-quality land cover class grade. Meanwhile, 66% of highly suitable habitats and 68% of moderate-suitability habitats occurred outside protected area boundaries. Targeted restoration of water body and swamp patches with high habitat quality contributions is needed to maintain Siberian crane populations. This work offers quantitative conservation strategies for this flagship species and provides theoretical support for spatial planning of the proposed Songnen Crane National Park.

## Simple Summary

To strengthen the protection of habitats on the Songnen Plain, ensure the survival of waterbirds, and protect their migration corridors, the Erlongtau River Basin was selected as the research area. A 33-year evaluation of waterbird habitat quality was conducted to analyze the factors influencing their changes. The ecological suitability of the flagship species, the Siberian crane, was assessed, and the spatial distribution of their land cover classess and associated influencing factors were clarified. The results indicate that the primary land use type for high-suitability habitat was dry farm located within and around the Momoge and Tumuji Nature Reserves and near areas connected to wetlands. With respect to high and moderate habitat suitability, 44% and 62% of the land cover classes were classified as “poor” in terms of habitat quality, and 66% and 68% of these habitat were located outside the reserve. To ensure the long-term conservation of Siberian cranes and consider the role of habitat quality in ecological sustainability, restoring wetlands that contribute substantially to habitat quality is essential. This study provides a reference for the conservation of endangered species and watershed ecological construction and offers scientific support and a theoretical foundation for the planning and development of Songnen Crane National Park.

## 1. Introduction

Globally, wetland ecosystems are degraded under climate change and anthropogenic interference, thereby continuously disturbing migratory waterbird migration channels and threatening the survival of endangered waders [1]. Quality reflects the overall ecological health and carrying capacity of wetland landscapes for general waterbird communities, while species-specific habitat suitability characterizes the degree to which an environment meets the unique ecological requirements of a single focal species. Importantly, these two metrics are not equivalent. The areas with highly comprehensive habitat quality for common waterbirds do not necessarily satisfy the specialized survival demands of flagship waders, leading to widespread spatial divergence between general ecological quality and species-specific usability [2,3,4]. Regional studies focused on the Songnen Plain wetland system have confirmed that long-term land cover alteration substantially reshapes habitat spatial patterns and affects waterbird habitat utilization [3,4,5].

Wetland patch area, spatial configuration, and landscape connectivity profoundly affect waterbird migration strategies and habitat utilization [2]. Regional wetland degradation in Northeast China is jointly driven by anthropogenic disturbances (wetland reclamation, agricultural expansion, and continuous water consumption) and climate changes (decreased precipitation and increased evaporation), collectively triggering severe wetland shrinkage, patch fragmentation, and functional degradation [1,2]. These changes disrupt food availability, movement corridors, and roosting conditions for migratory waterbirds. Although previous studies have documented general wetland degradation trends in this region, systematic watershed-scale quantitative analyses of how multi-source land use conversions drive long-term habitat quality dynamics are lacking. Habitat suitability modeling provides an effective tool with which to clarify species-environment interactions, identify core habitats, and support refined conservation management [6,7,8].

Conservation gap analysis is a core framework for identifying unprotected but ecologically critical habitats for endangered migratory species. Existing protected-area networks are often delineated based on general wetland coverage rather than species-specific habitat demands, resulting in frequent mismatches between reserve boundaries and actual species distribution [7,8,9,10]. For migratory waterbirds with long-distance migration, stopover sites outside reserves often undertake critical energy supplementation functions. Therefore, combining general quality evaluation and species-specific suitability mapping is a necessary approach through which to accurately identify conservation gaps and assess the effectiveness of existing protected area systems.

The Songnen Plain is located at the intersection of multiple East Asian migratory flyways, serving as an irreplaceable stopover site for vulnerable waterbirds. As a typical independent hydrological unit in the northeastern Songnen Plain, the Erlongtao River Basin determines regional wetland connectivity, hydrological cycles, and human disturbance patterns, representing the most appropriate spatial scale for assessing local waterbird habitat changes and conservation efficiency. In recent decades, cumulative anthropogenic and climatic pressures have severely weakened wetland ecosystem functions within this basin [1]. Two core mechanism groups driving habitat changes should be analyzed: land use transition processes dominated by wetland reclamation and farmland expansion and climate–hydrological variations including precipitation reduction and evapotranspiration increase. Classified as Critically Endangered (CR) by the IUCN Red List, the Siberian crane (*Leucogeranus leucogeranus*) migrates between Russian breeding grounds and Poyang Lake wintering habitats [6,10]. As a typical flagship and umbrella species for temperate inland wetland ecosystems, the protection of Siberian crane habitats can benefit sympatric waterbird communities and entire wetland landscapes. Notably, field surveys confirm that post-harvest farmland residues have become a major food resource for migratory Siberian cranes, forming a key ecological contradiction that, increasingly, Siberian cranes’ agricultural habitats feature low overall habitat quality and poor long-term sustainability, while high-quality natural wetlands fail to fully meet current foraging requirements. Most migratory Siberian crane populations occur outside protected boundaries during stopover periods, and the existing Chinese reserve network exhibits prominent conservation gaps and insufficient targeted protection capacity [11,12,13,14,15]. Hence, targeted habitat conservation for migratory Siberian cranes in this basin is strategically significant for regional wetland ecological restoration.

Despite accumulated wetland ecological research in the Songnen Plain, three key problems remain unresolved at the watershed scale: (1) the long-term spatiotemporal evolution characteristics of waterbird habitat quality over 33 years and the quantitative driving contributions of different land use conversions remain unclear; (2) the spatial mismatch mechanism between basin-wide general habitat quality and Siberian crane-specific habitat suitability has not been systematically quantified; and (3) the spatial distribution, area proportion, and land use composition of unprotected suitable habitats for Siberian cranes are undefined, restricting refined wetland management and conservation planning. Based on these problems, this study adopts 33-year land use datasets (1990–2023), the InVEST habitat quality model, and the MaxEnt species suitability model to clarify long-term habitat dynamics, driving mechanisms, and conservation gap patterns of Siberian cranes in the Erlongtao River Basin. In this study, the divergent relationship between general habitat quality and species-specific suitability will be clarified, and the core conservation contradiction between wetland degradation and agricultural habitat dependence will be revealed. It hopes to provide a scientific support for future waterbird conservation and regional ecological sustainability.

## 2. Materials and Methods

### 2.1. Study Area

**Figure 1:**
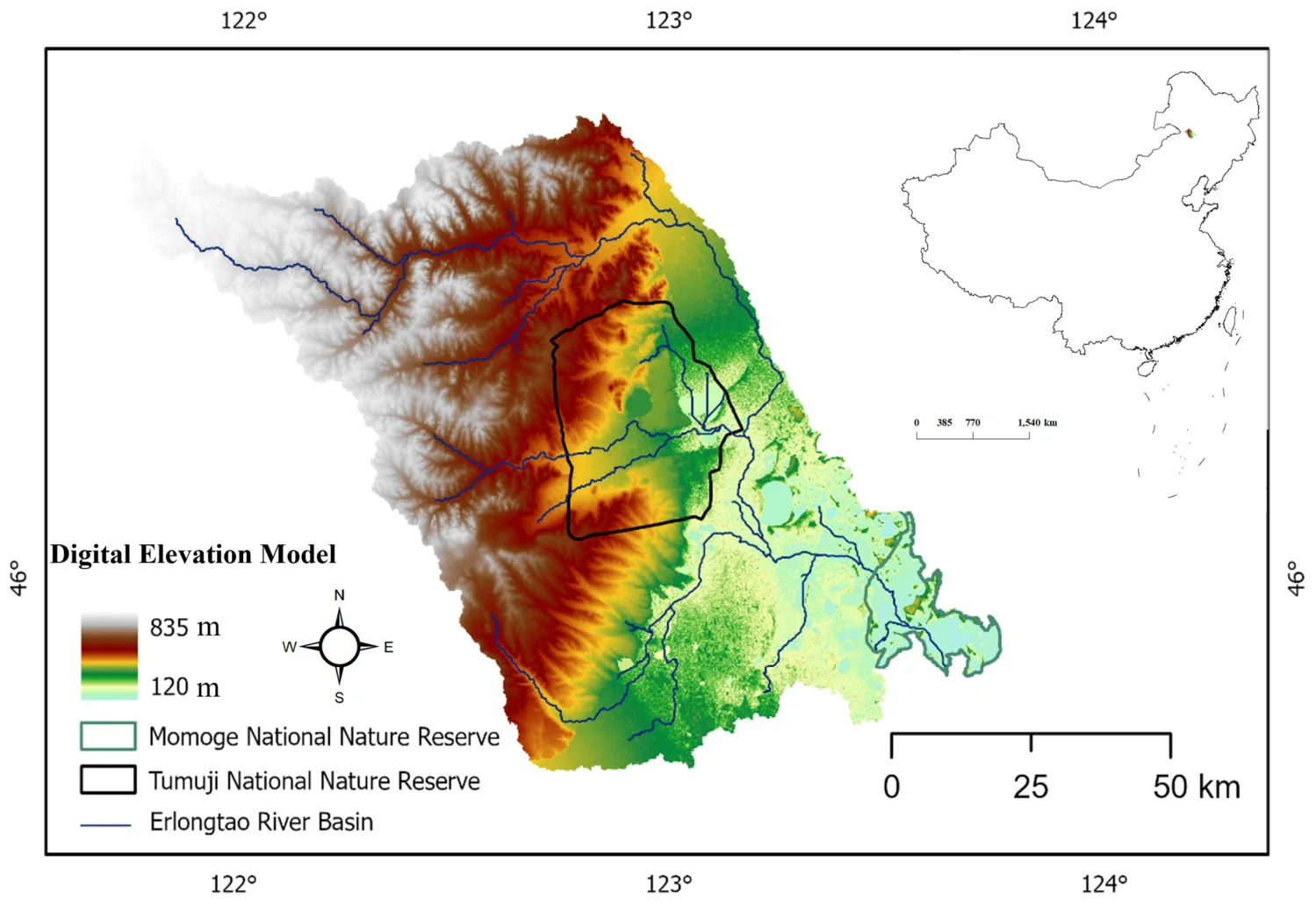
The study area.

The Erlongtao River Basin is in the semiarid region of the Songnen Plain in northeastern China. It has a temperate continental monsoon climate and spans a total length of 178.2 km. The terrain is relatively high in the northwest and low in the southeast, with the characteristics of a typical seasonal watercourse (Figure 1). The flat terrain in the middle and lower reaches causes the river to spread out, resulting in the formation of extensive wetlands and large agricultural areas, which provide essential natural and ecological resources for waterbird stopovers. The basin flows through the Inner Mongolia Tumuji National Nature Reserve and Jilin Momoge National Nature Reserve. In recent years, approximately 4000 migrating Siberian cranes have been recorded in this basin, accounting for approximately 75% of the global wild Siberian crane population. The Siberian cranes forage primarily in the wetlands and dry farm of the region. In wetland land cover classes, Siberian cranes mainly feed on the corm of *Bolboschoenus planiculmis*. In dry farm land cover classes, the primary food source is the remaining leftover corm after harvest [16].

### 2.2. Data Sources

#### 2.2.1. Field Survey of Siberian Cranes and Their Land Cover Classes

The distribution of Siberian cranes and the characteristics of their land cover classes were surveyed using sample point interpolation and line transects (the sample line method and the sample point method) during the spring and autumn migration stopover periods from October 2020 to May 2024. Once the Siberian cranes were located, the observations were immediately conducted from high terrain using 8× binoculars and a 20 × 60 monocular telescope. Data on human disturbance, geographic coordinates, and land cover classes selection were recorded.

Field transects were laid out along rural roads and national highways near the tidal flats of the study area, with priority given to waters and ponds that are potential land cover classes for Siberian cranes. Researchers walked along the routes and continuously surveyed the surroundings with telescopes. Once flocks of Siberian cranes were found, we set up fixed sampling points for focused observation. Instead of predefining transects with fixed total lengths, this study adopted an adaptive route patrol method. A total of 108 fixed observation points were confirmed across the whole basin. Field investigations were conducted during the spring and autumn migration stopover stages of Siberian cranes from October 2020 to May 2024. Two fixed observation periods were arranged each day—06:00–09:00 in the morning and 15:00–18:00 in the afternoon—with each site observed continuously for no less than 40 min. An 8× binocular and a 20–60× monocular telescope were adopted for panoramic scanning and focal tracking of individuals, with an effective observation distance of 1–2 km. Real-time GPS coordinates, the number of Siberian cranes, behavioral types (foraging, resting, migration), land cover classes, human disturbance types (agricultural activities, road traffic, construction), daily temperature and aquatic hydrological conditions were recorded synchronously in the field. A unified recording standard was followed throughout all surveys. Two investigators recorded data simultaneously for cross-checking, and abnormal outlier points were removed to complete data quality control.

#### 2.2.2. Landscape Pattern Analysis

To reveal the characteristics of land use structural changes in the study area, land use data were selected for eight periods (1990, 1995, 2000, 2005, 2010, 2015, 2020 and 2023) in this study. This is because they cover the key phases of agricultural reclamation, wetland conservation, and ecological engineering in the Songnen Plain, allowing us to characterize long-term land cover dynamics over more than 30 years. The 5-year time interval was adopted for the period 1990–2020, consistent with the regular release cycle of national land use remote sensing datasets in China. A shorter 3-year interval (2020–2023) was set to obtain rapid short-term changes in wetlands and farmlands driven by recent water conservancy projects. The data were obtained from the Resource and Environment Science and Data Center of the Chinese Academy of Sciences (http://www.resdc.cn). According to the official metadata and dataset manual released by the data center, all multi-temporal land use raster products adopted in this study have an overall classification accuracy higher than 89% and a Kappa coefficient greater than 0.83. The confusion matrix and precision assessment results are provided by the official data supplier; thus, no extra independent accuracy verification was conducted in this study. The nine land use categories were defined based on three standards: the official national land use classification system of China, the interpretability of remote sensing images, and the land cover class selection characteristics of Siberian cranes in the Songnen Plain wetland ecosystem. Nine land use categories—paddy fields, dry farms, forestland, grasslands, water areas, shallow areas, construction, unutilized land, and swamps—were identified by mask extraction and reclassification. The land use transfer matrices were calculated using spatial overlay analysis in ArcGIS 10.8 to examine the dynamic evolution processes of different land use types between consecutive periods. To standardize the expression of land cover classes and make the classification rules clear, the identification standards of the nine land use categories were defined in Table S1.

#### 2.2.3. Habitat Quality Assessment for Waterbirds

The InVEST Habitat Quality module can efficiently quantify spatial–temporal variations in habitat quality at the watershed scale and has been widely applied for wetland-waterbird conservation assessments in similar temperate wetland systems [17,18]. The model links land cover patterns with multiple anthropogenic and natural threat layers to produce continuous habitat quality outputs without requiring detailed species abundance data. Therefore, the InVEST model was used to calculate the spatial distribution of habitat quality in the study area during the eight periods of 1990, 1995, 2000, 2005, 2010, 2015, 2020, and 2023.

(1) Selection of threat factors

In this study, construction, unutilized land, and roads were identified as threat factors, and their spatial distributions were represented using GIS raster data. The influence distances, threat weights, and decay types of the threat sources were determined based on the InVEST model, field survey data, and previous studies [19]. Construction land and roads represent persistent direct human disturbances, while unused land reflects degraded land surfaces in this semi-arid basin (Table 1).

(2) Sensitivity of habitat to threat factors

The distribution points of Siberian cranes and land cover types of the study area were imported into the MaxEnt model to obtain the habitat suitability of Siberian cranes for different habitat types, as well as the contribution value of each habitat to the suitable habitat distribution of the species. Habitat suitability was classified according to the degree of influence of human activities on land use types, ranging from 0 to 1. A value of 0 means that this land type is not a suitable habitat [20]. Referring to previous studies and the InVEST model user guide, the sensitivity of each threat factor in the study area was assigned to quantify its impact on habitat suitability (Table 2).

(3) Habitat quality calculation by the InVEST model

The habitat quality module of the InVEST model was used to evaluate the habitat quality in the study area based on Table 1 and Table 2. The assessment results range from 0 to 1, where a value closer to 1 indicates lower levels of human disturbance, higher habitat quality, and greater biodiversity levels.

(4) Calculation of contribution rate of land use transition

Taking the two-phase land use change datasets derived via geographic information mapping as input raster data, the raster calculator tool in ArcGIS was used to generate raster layers representing habitat quality changes between every two adjacent periods, which were taken as the assigned input raster features. The Zonal Statistics as Table tool of ArcGIS was used to calculate the average habitat quality variation corresponding to each land conversion type. Finally, the contribution rate of each land use transition to habitat quality was calculated by the following formula.

(1) $$R_{ij}=\frac{(H_{j}-H_{i})S_{i}}{s_{t}}$$

where *R_ij_* is the contribution rate to habitat quality when land type *i* is converted to land type *j*; *H_i_* is the habitat quality index in the initial stage of the research period for the corresponding land patches; *H_j_* is the habitat quality index in the terminal stage of the research period for the corresponding land patches; *S_i_* is the total area of patches undergoing the *i* → *j* land conversion; and *S_t_* is the total land area of the entire study watershed.

#### 2.2.4. Habitat Suitability for Siberian Cranes

Twelve environmental factors were selected based on field investigations and the InVEST model in this study (Table 3). Euclidean distance analysis was conducted for the accessibility factors to generate raster maps of the distance factors. All the raster data were projected to the spatial coordinate system WGS_1984_UTM_Zone_51N with a spatial resolution of 30 m × 30 m. Mask extraction was applied to the raster data to ensure spatial consistency across the dataset.

Species distribution data (CSV format) and environmental data (ASCII format) were input into the Maxent model to construct a distribution model for the Siberian crane. The performance of the model was evaluated using the area under the curve (AUC), and the importance of environmental factors was tested using the jackknife method [6,21]. The area under the curve (AUC) was adopted as a quantitative indicator for evaluating model predictive performance, with values ranging from 0 to 1. The output suitability values ranging from 0 to 1 were further divided into four grades in ArcGIS.

To reduce random errors, the MaxEnt model was run for 100 replicates. Spatial autocorrelation of species points was eliminated by setting a minimum Euclidean distance of 1.2 km between independent crane occurrence records. Some 75% of field distribution points were randomly selected as training data, and the remaining 25% were used as validation data. Model performance was comprehensively assessed using training AUC, test AUC, omission rate, and the jackknife test for variable importance and partial ROC curves, rather than relying solely on single AUC values. To resolve temporal mismatch between 2020–2024 field survey data and multi-temporal land use layers, the latest 2023 land use raster was uniformly used as the land cover predictor variable. Land use type was only retained as an auxiliary factor, with topography, climate, vegetation, and hydrology as core predictors to avoid circular reasoning.

#### 2.2.5. Characteristics of Habitat Suitability for Siberian Cranes and Gap Analysis

The suitability levels of the habitat derived from the Maxent model were extracted by mask. Land use and habitat quality data were used as input data, and ArcGIS was used to determine the land use/land cover and habitat quality at each suitability level. The resulting habitat suitability layers were subsequently used as “input features”, whereas the Tumuji and Momoge National Nature Reserve data served as “output features” for clipping. The area of each suitability level within each reserve was calculated. The conservation gap for each suitability level was determined by subtracting the area within the reserves from the total suitable habitat area in the study area.

### 2.3. Statistical Analysis

Statistical analysis was performed by SPSS 26.0. A one-way ANOVA was used to analyze inter-annual differences of land use area metrics and habitat quality values.

## 3. Results

### 3.1. Characteristics of Land Use Changes

**Figure 2:**
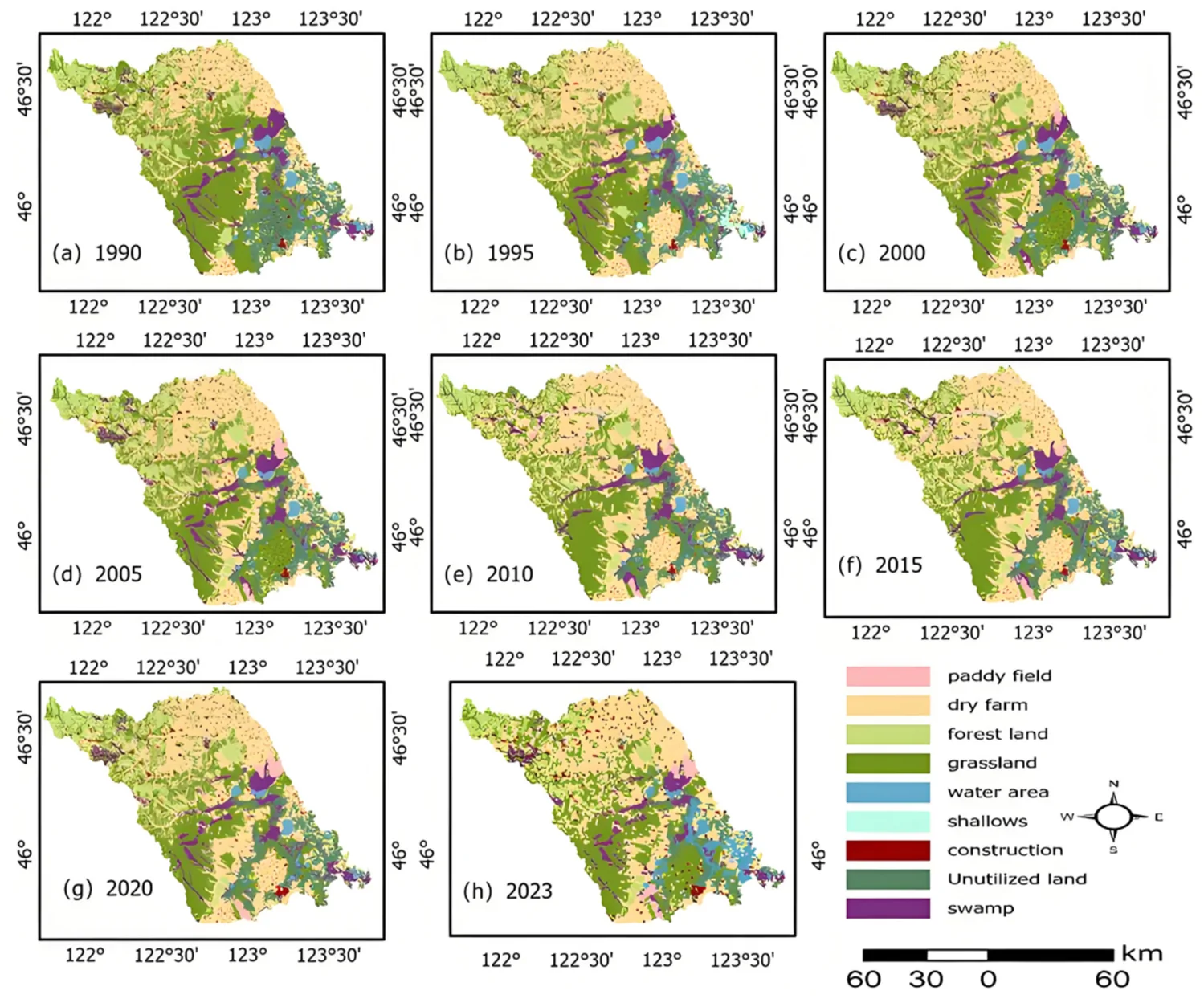
Distributions of land use types.

Land use classification outputs for eight time points (1990, 1995, 2000, 2005, 2010, 2015, 2020, and 2023) are illustrated in Figure 2. Dry farmland and grassland dominated the basin, jointly accounting for approximately 71% of the total watershed area across the 33 year study period. Water bodies, shallows, and built-up land occupied relatively small proportional shares.

Table 4 summarizes the areal extent of each land use category. Percentage change and net areal difference between 1990 and 2023 were calculated to statistically compare land use dynamics. Dry farm exhibited the largest net areal increase, expanding from 2443.90 km^2^ to 3092.20 km^2^. Paddy field increased from 1990 to 2015 and declined thereafter. Forestland showed an overall increasing trend with a temporary decrease between 1995 and 2000. Grassland experienced a net reduction, with major areal losses observed during 1990–1995 and 2010–2015. Water area generally decreased, interrupted by short-term increases in 2005–2010 and a sharp rise in 2023. Construction land decreased from 1990 to 2015 and peaked in 2020. Unutilized land decreased overall despite minor growth during 2005–2010. Statistical comparison of land use areas indicated that arable land (paddy and dry farmland), forestland, and construction land grew in area, whereas grassland, unutilized land, and swamps experienced net area loss during the study period. Shallows showed the smallest net change among all land use classes.

### 3.2. Changes in Habitat Quality in the Study Area

Area statistics for four habitat quality grades across 1990–2023 are shown in Table 5. Across all eight observation years, the area rank of habitat quality grades consistently followed a relatively poor > poor > good > excellent order. The “relatively poor” grade occupied the largest proportion of the watershed, whereas “good” and “excellent” habitats constituted minor fractions of the total area.

For the “poor” grade, the net area change from 1990 to 2023 reached 684.08 km^2^ (−50.5%). The “relatively poor” habitat exhibited a net increase of 7.4% over 33 years, expanding from 5751.27 km^2^ (1990) to 6175.38 km^2^ (2023). The “good” habitat experienced a net increase of 43.7%, declining from 750.20 km^2^ in 1990 to the minimum of 690.05 km^2^ in 2010, followed by a substantial rise to 1077.61 km^2^ in 2023. The “excellent” habitat showed a net decrease of 11.0%, fluctuating between a maximum of 670.38 km^2^ (2000) and a minimum of 533.45 km^2^ (2015).

**Figure 3:**
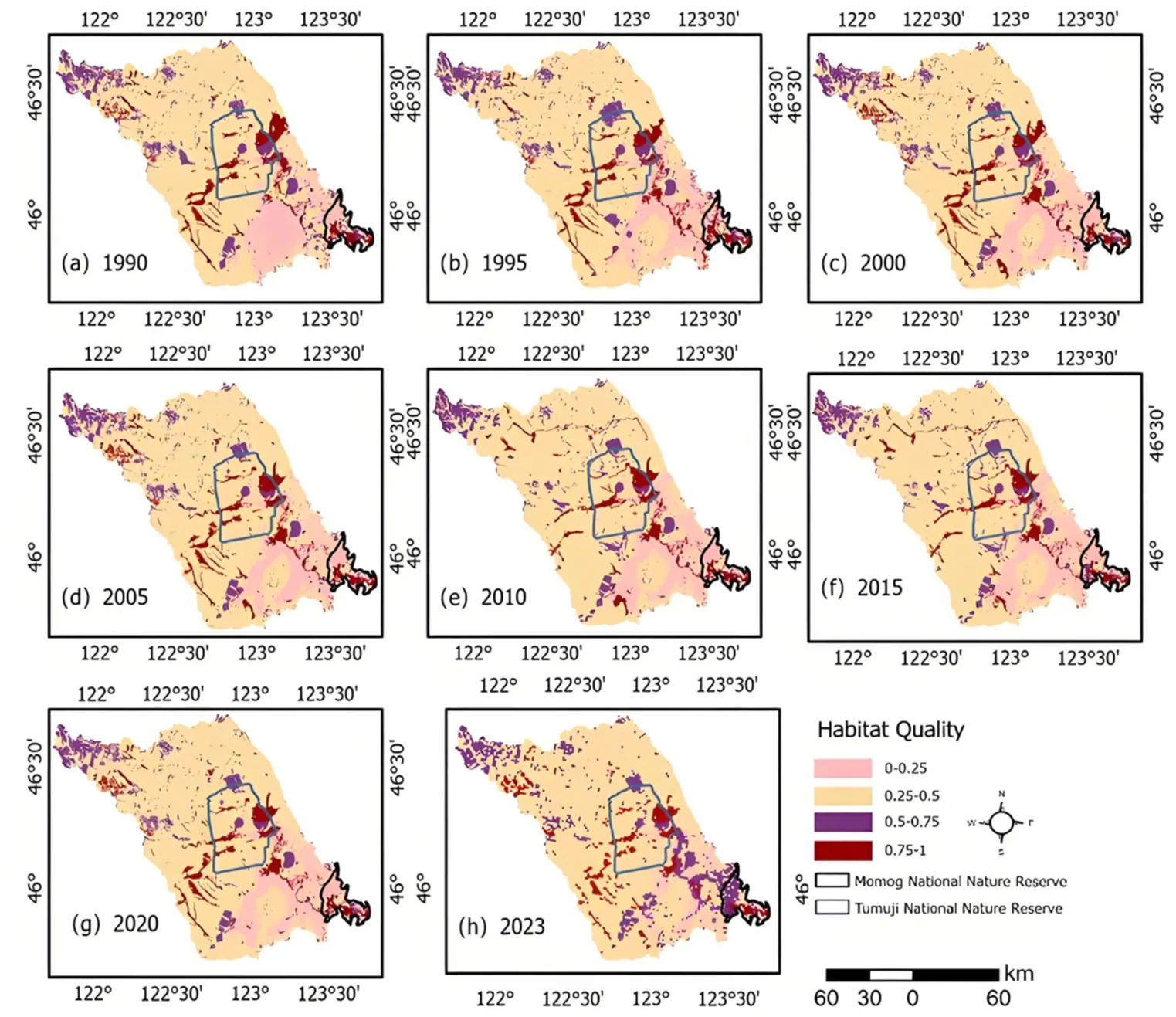
Habitat quality distributions from 1990 to 2023.

Spatially, habitat quality presented a distinct spatial pattern of high values in the southeast and low values in the northwest (Figure 3). Excellent- and good-grade habitats were predominantly distributed over wetlands, shallows, grasslands and open water within low-elevation plains. Poor and relatively poor habitat mainly corresponded to built-up areas and unutilized land, including towns and villages. The combined area of poor and relatively poor habitat reached a high point in 2010, concurrent with the maximum extent of dry farmland and unutilized land and the contraction of water body and wetland patches.

### 3.3. Contribution of Land Use Change to Habitat Quality

**Figure 4:**
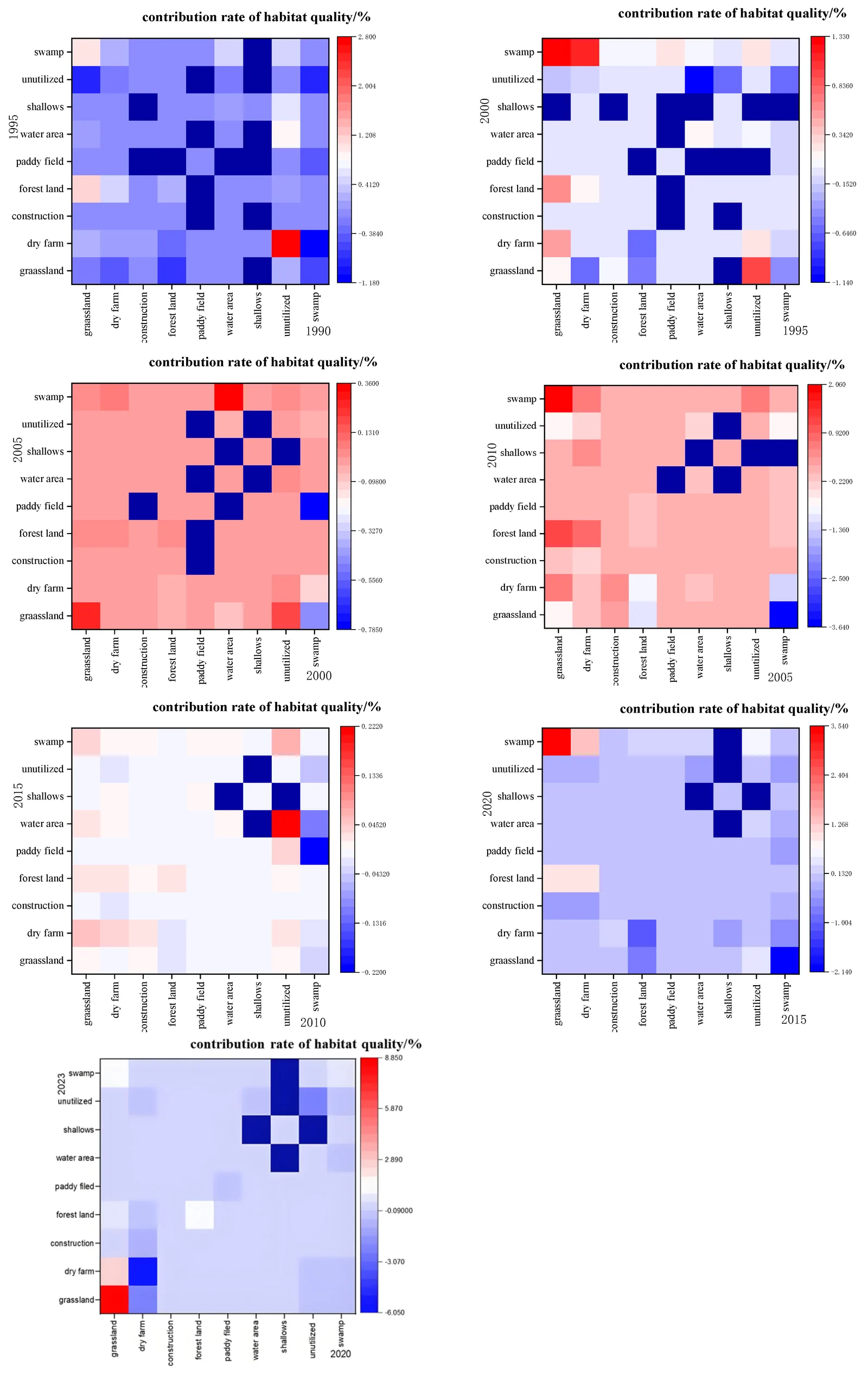
Contribution rate of habitat quality in the study area from 1990 to 2023.

An analysis of the spatial variation trend of habitat quality in the study area from 1990 to 2023 revealed that the overall habitat quality in the study area has remained relatively stable over the past 33 years and has not experienced significant fluctuations (Figure 4). An analysis of the spatiotemporal changes in land use in the study area revealed that the ecological environment of waterbirds in the study area changed substantially from 1990 to 2023.

In terms of the contribution rate of land use transfer to habitat quality from 1990 to 2023, shallows were mainly converted into unutilized land, forestland, and dry farm, whereas swamps were mainly converted into unutilized land, dry farm, paddy fields, and grassland. Water areas were mainly transformed into unutilized land, swamps, and shallows. Grasslands were mainly transformed into dry farms and swamps. Unutilized land was converted primarily into water areas, whereas paddy fields, dry farm, developed land, and forestland increased. Swamp and dry farm were the main transfer types for paddy fields, whereas grassland was the main transfer type for dry farm and forestland. Overall, arable land, forestland, and construction land expanded, while grassland, unutilized land, water bodies, and swamps shrank. From 1990 to 1995, unutilized land was converted into water area, grasslands were converted into swamps, and grasslands were converted into forestland. The conversion between these three types of land contributed substantially to the quality of the habitat. The conversion of unutilized land into grassland substantially contributed to the qualities of the habitat from 1995 to 2000 and from 2000 to 2005. The conversion of unutilized land and dry farm into swamps substantially contributed to habitat quality from 2005 to 2010. The transformation of grassland into swamps substantially contributed to habitat quality from 2015 to 2020. The conversion of grassland into dry farm substantially contributed to habitat quality from 2020 to 2023.

### 3.4. Suitable Habitat and Conservation Gaps for Siberian Cranes

**Figure 5:**
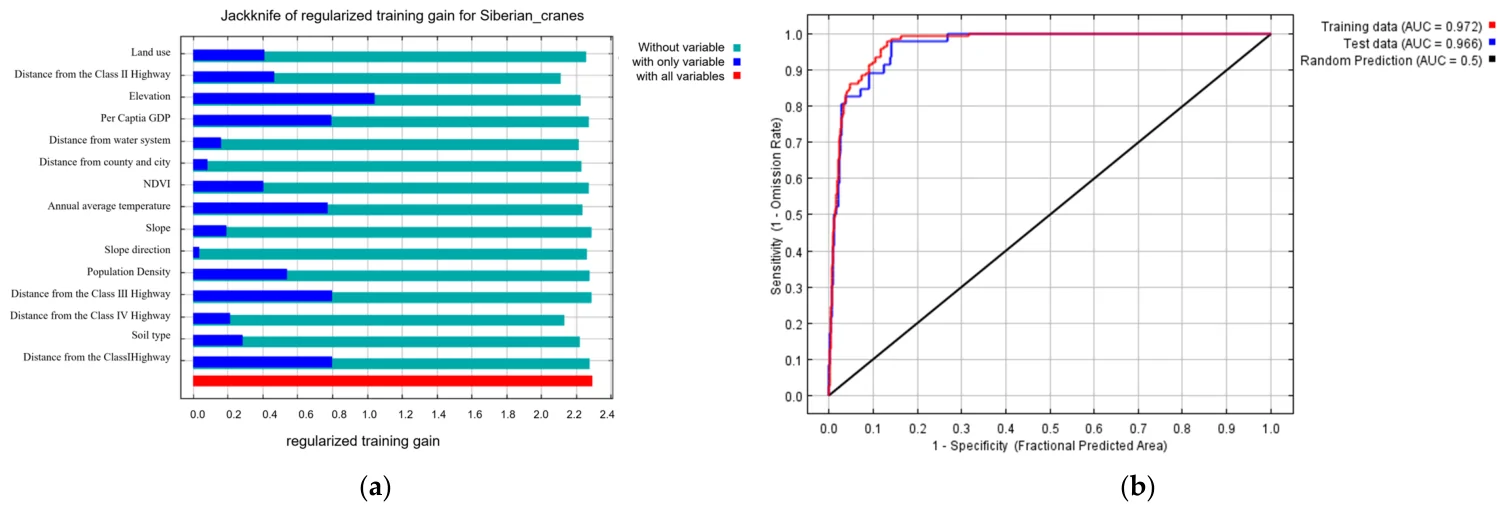
Jackknife of regularized training gain for Siberian_cranes (**a**) and ROC curve and AUC value of the MaxEnt model for Siberian cranes (**b**).

The results of the jackknife test and ROC curve are shown in Figure 5. The jackknife test indicated that elevation contributed the most to the training gain of the model when each environmental variable was used independently. The average AUC value of the test dataset for this model was 0.972 (AUC > 0.9). According to the model evaluation criteria proposed by Phillips et al., this value indicates high model-fitting performance, and the prediction results are reasonable and reliable for assessing the habitat suitability of Siberian cranes in the study area [21]. The permutation importance analysis of variable indicated that the elevation contributed most to the model gain (39.5%) (Table 6).

The response curves of habitat suitability for Siberian cranes to various environmental factors are shown in Figure S1, which reflect the variation characteristics of habitat suitability probability along environmental gradients. The habitat suitability of Siberian cranes decreased markedly with the increase in elevation, slope, and population density. The highest habitat suitability occurred in areas close to water systems, and there was an optimal range of annual mean temperature for the survival of Siberian cranes.

**Figure 6:**
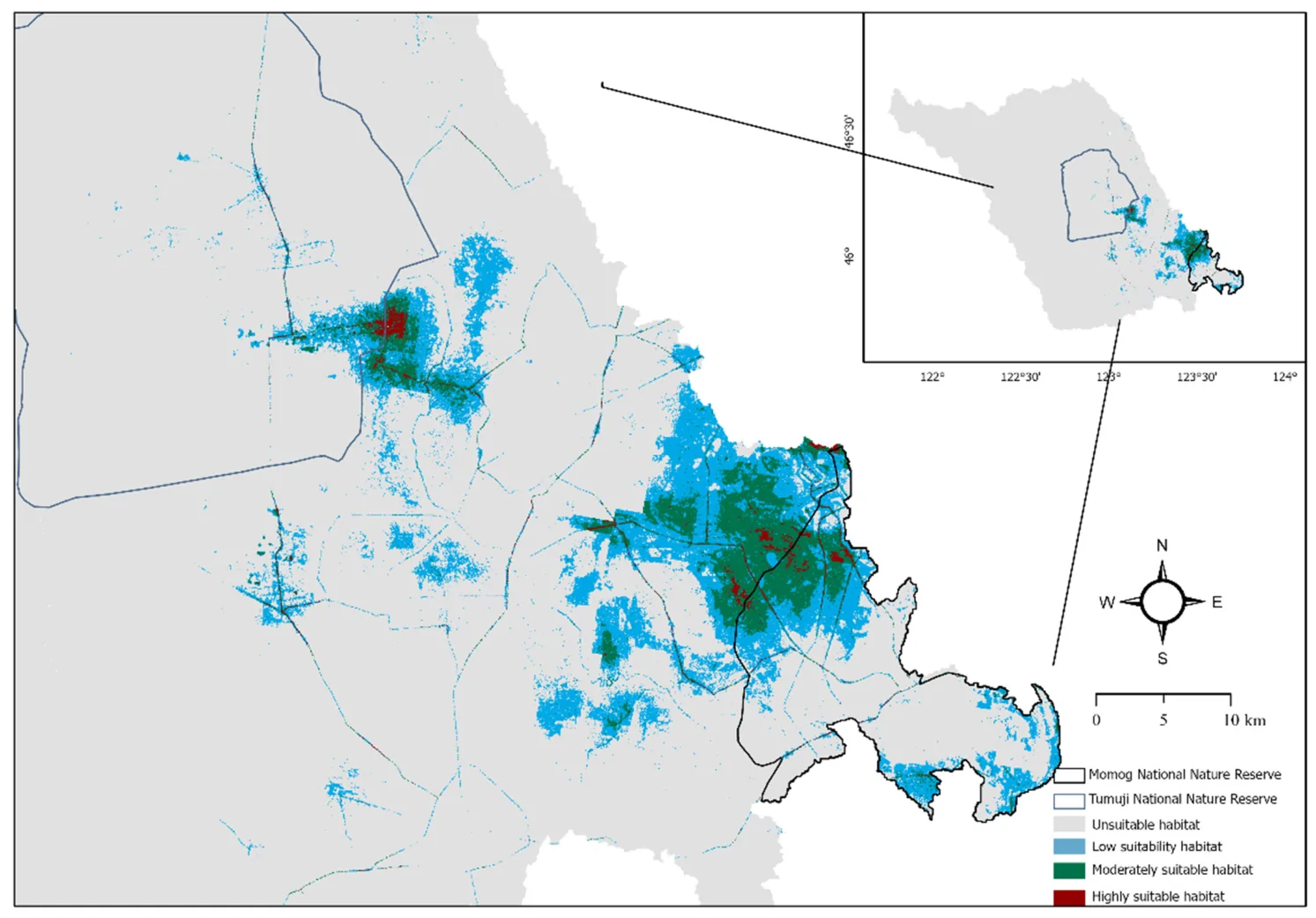
Spatial distributions of highly suitable habitat.

The unsuitable habitat is located mainly in the northwestern part of the study area, whereas high-, moderate-, and low-suitability habitats are distributed primarily within and around the Momoge and Tumuji Nature Reserves. These areas were characterized by numerous wetlands and high hydrological connectivity (Figure 6).

**Figure 7:**
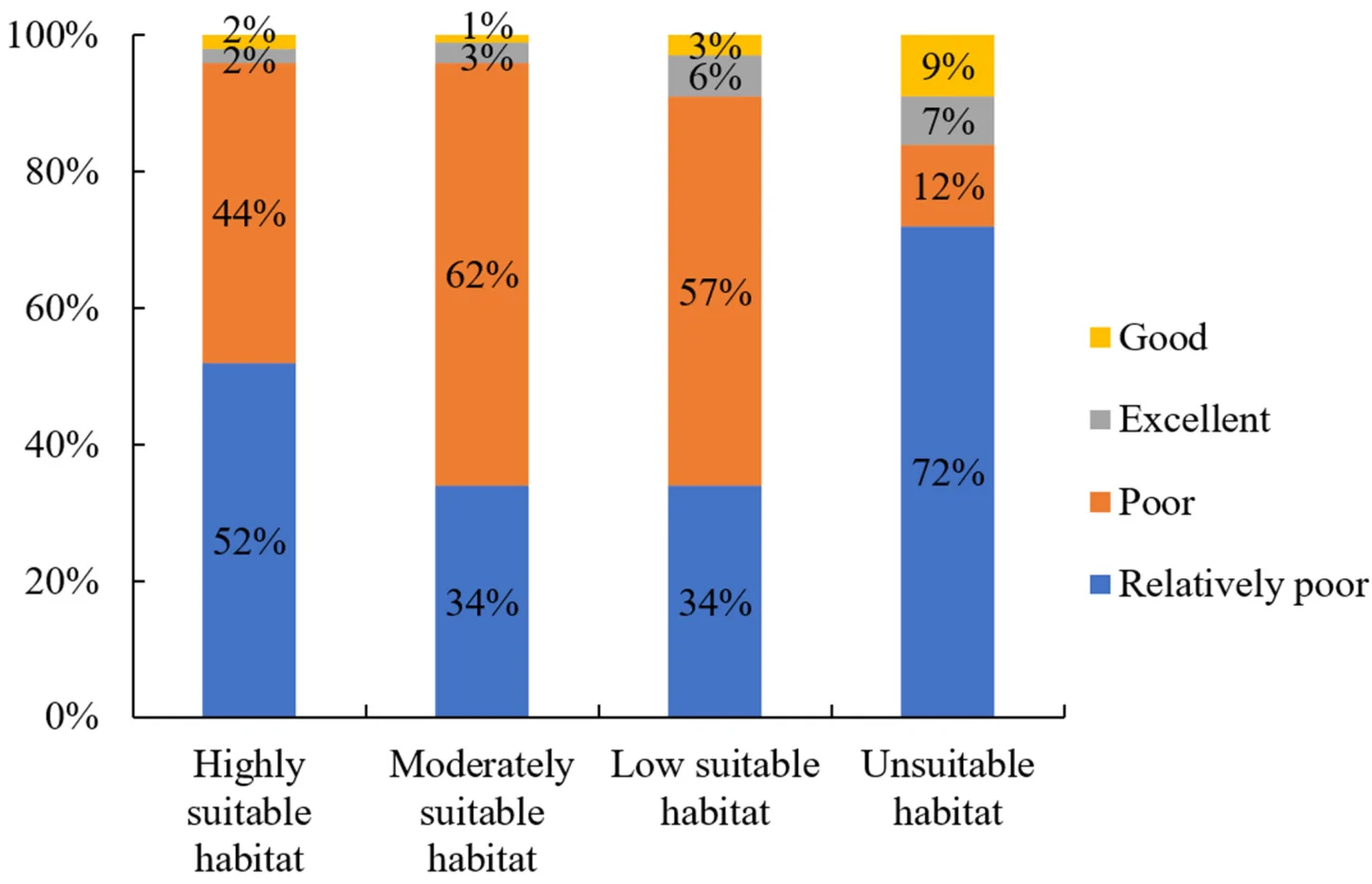
Proportions of different-suitability habitats for four habitat qualities.

This study revealed that 47.97%, 31.79%, and 28.04% of high-, moderate-, and low-suitability habitats, respectively, were composed of dry farm (Table 7). Moreover, 44% of the high-suitability habitats and 62% of the moderate-suitability habitats were classified as “poor” in terms of habitat quality (Figure 7).

**Figure 8:**
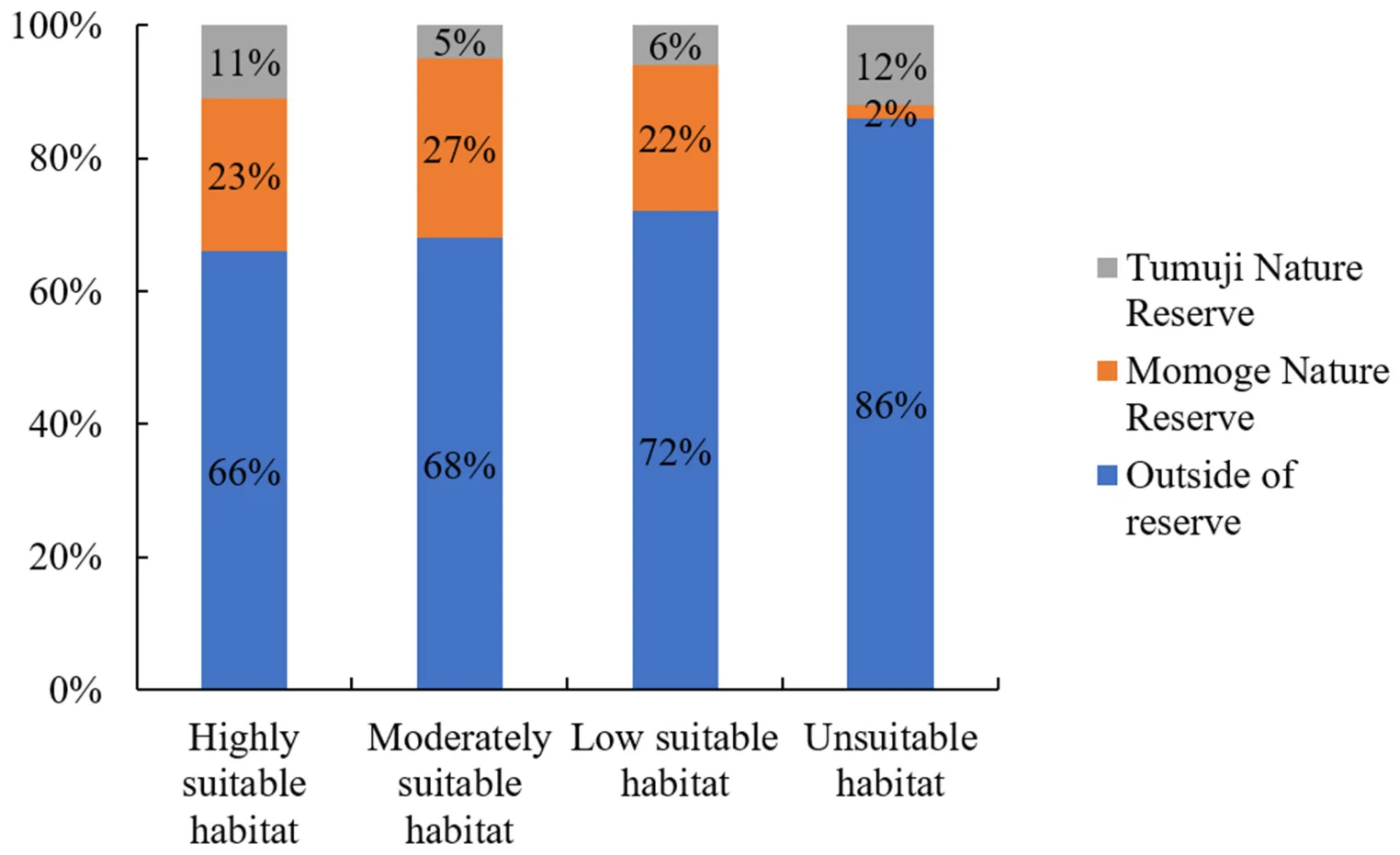
Percentages of different-suitability habitats inside and outside the reserve.

Among the high-suitability habitats, 11% were located at the Tumuji Nature Reserve, 23% were within the Momoge Nature Reserve, and 66% were outside of protected areas. With respect to moderate-suitability habitats, 5% were located at the Tumuji Nature Reserve, 27% were within the Momoge Nature Reserve, and 68% were outside of protected areas (Figure 8). In total, high-, moderate-, and low-suitability habitats cover 14.99, 116.37, and 300.05 km^2^, respectively, of which 9.86, 79.06, and 216.24 km^2^ are located outside the two national nature reserves (Table 8).

## 4. Discussion

### 4.1. Land Use Changes

The land cover classes of Siberian cranes in the study area changed substantially from 1990 to 2023. Analysis of the land use (1995–2000) revealed that most of the water areas were transformed into other land type. This variation was driven by a warming and drying climate, reduced precipitation, high evaporation rates, and hydraulic projects that diminished river and lake flooding and created favorable hydrological conditions for farmland reclamation [21,22]. From 2000 to 2010, water bodies and swamps continued to decline, whereas paddy fields and dry farmland expanded. Annual precipitation decreased continuously after 2000. To meet agricultural and domestic water needs, reservoirs were constructed for the Tao’er and Erlongtao Rivers. These reservoirs caused prolonged flow interruptions in the Erlongtao River and reduced runoff in the Tao’er River, leading to a prolonged dry period [23]. Additionally, rapid agricultural expansion and unregulated wetland development increased groundwater demand, altering groundwater-wetland interactions and leading to wetland salinization. Therefore, unutilized land (bare land and saline-alkali land) increased during this decade.

To strengthen national food security, major water conservancy projects, such as the River–Lake Connectivity Project, were constructed in western Jilin Province from 2010 to 2020. These projects transfer the excess water from the Nen River (the largest tributary of the Songhua River), Tao’er River, and Huolin River into natural lakes and wetlands via an interconnected water network. This approach not only promoted the recovery of forests and wetlands but also converted large areas of saline-alkali land into arable land through a “rice cultivation for alkalization control” model. As a result, farmland (paddy and dry fields) gradually expanded, whereas unutilized land decreased.

The reduction in swamps and shallow areas has weakened hydrological regulation and biodiversity within the watershed. The fragmentation of wetlands reduces their ability to support the land cover classes and foraging of migratory waterbirds, posing a threat to the stability of regional ecosystems. The rapid expansion of paddy fields and dry land has met demand for food production, but it has encroached on natural land, such as water bodies, beaches, and swamps, and disrupted the natural ecological pattern in the region, especially in the southeastern and central southern parts, where land use conflicts between wetlands and farmland are particularly evident.

### 4.2. Changes in Habitat Quality

Land use change has resulted in spatial and temporal heterogeneity in the distribution of habitat quality [24]. Good- and excellent-quality habitat are associated with land use types such as wetlands, tidal flats, and water bodies. These land cover classes have extensive water systems, abundant wetland resources, and numerous marshes, providing suitable habitat and food resources for species. “Poor” and “relatively poor” habitats are affected primarily by human disturbances such as land salinization, agricultural land reclamation, and straw burning.

Water bodies, beaches, and swamps substantially contribute to the habitat quality of Siberian cranes, whereas construction land and unutilized land threaten the survival of waterbirds. The cultivation of wasteland and land salinization has caused drastic changes in wetland landscapes, resulting in changes in land use type of the study area. These changes are related to the destruction of grassland, deforestation, wasteland and the construction of infrastructure, as well as the protection and construction of basic farmland by the government. Water conservancy engineering and climate conditions in the research area lead to water level fluctuations, which are the main factors affecting the coupled relationship between waterbirds and land cover classes, causing environmental problems such as habitat fragmentation and affecting landscape patterns [25]. The fragmentation of landscape patterns and the loss and degradation of land cover classes have led to a decline in waterbird populations [26,27]. The degradation of habitat quality is consistent with the large-scale loss of natural wetlands, which serve as land cover classes for waterbirds.

The driving mechanism of changes in habitat quality is multifactorial and comprehensive. Both human and natural factors, such as elevation and precipitation, influence changes in habitat quality [28,29]. In addition, the elevation of the study area increased from southeast to northwest, and the areas with poor habitat quality for many years were mostly distributed throughout the northwest, which is consistent with the results of this study. The establishment of nature reserves promotes effective protection and continuous management, reducing adverse land cover classes and human disturbances in and around the region. As a result, excellent-quality land cover classes are located primarily within and around the Tumuji and Momoge Nature Reserves. In the study area, the habitat quality decreased progressively with increasing elevation. Consecutive drought years resulted in poor hydrological conditions from 2000 to 2010, and rapid agricultural development and unregulated wetland exploitation caused a decline in excellent land cover class areas during this period. However, the development of major water conservancy projects from 2010 to 2023 promoted wetland restoration, resulting in an increase in “excellent” land cover class areas during this period.

### 4.3. Characteristics of Suitable Habitats for Siberian Cranes

The area of suitable habitat for Siberian cranes in the northwestern part of the study area is relatively small because of the relatively high elevation and predominant land use type (forestland interspersed with villages), which are unfavorable for Siberian crane stopovers. The suitable habitats are primarily concentrated in the southeastern part of the study area. The dominant land use types in this area are dry farms and water areas. Dry farms account for 47.97%, 31.79%, and 28.04% of the high-, moderate-, and low-suitability habitats, respectively, whereas the combined areas of water and swamps account for 2.15%, 3.57%, and 8.79%, respectively. Wetlands constitute a small proportion of suitable habitats due to rising water levels in the region caused by recent hydraulic projects and climatic conditions. Shallow-water areas suitable for Siberian crane stopovers have disappeared, and large deep-water wetlands are not conducive to Siberian crane stopovers. The study area has extensive dry farms, where mechanically harvested residual crops left on the soil surface or buried in the topsoil, such as corn kernels and peanuts, provide abundant food resources for Siberian cranes and supply their energy needs during migration. As a result, the survival strategy of Siberian cranes has transferred toward dry farm that can provide more food resources.

Although dry farms constitute a major part of suitable land cover classes, not all dry farms are suitable for Siberian cranes. Extensive dry farms are present in the northwestern part of the study area. However, the lack of surrounding water systems makes these land cover classes rare for Siberian crane stopovers. In contrast, the dry farm in the southeastern Tumuji Nature Reserve is surrounded by marshes. This interconnected structure provides access to water while reducing dependence on original wetland land cover classes, contributing to the return of Siberian cranes to wetlands for rest at night and making these dry farms more suitable. Therefore, the proximity of dry farms to water systems is crucial for determining the suitability of dry farms for Siberian cranes.

The primary land use type for habitats suitable for Siberian cranes is dry farm, which negatively impact habitat quality. As a result, the habitat quality of land suitable for Siberian cranes is relatively poor. Poor-quality habitats accounted for 44%, 62%, and 57% of the high-, moderate-, and low-suitability habitats, respectively. However, the original wetlands of Siberian cranes have a positive influence on habitat quality. Water resources in wetlands shape their structure and characteristics, which regulate landscape pattern changes, ultimately affecting the population structure, distribution, and stopover numbers of waterbirds. Wetlands play crucial roles in maintaining biodiversity, regulating climate, conserving water resources, purifying water and mitigating floods [30,31]. Therefore, to ensure the sustainability of Siberian crane habitats, efforts should be focused on restoring their original wetland to encourage cranes to return to wetlands for foraging.

### 4.4. Conservation Gaps in Suitable Habitat

The spring and autumn migration periods of Siberian cranes are long and represent the most perilous periods for the species. Unsuccessful migrations could result in life-threatening risks for Siberian cranes, potentially impacting population reproduction [32]. Therefore, the conservation efficiency of migration stopover sites is critically important. The Erlongtao River Basin has significant conservation gaps for Siberian cranes. Approximately 66%, 68%, and 72% of the high-, moderate-, and low-suitability habitats, respectively, are located outside protected areas. Siberian crane migration periods are the same as those of the spring and autumn farming seasons in the basin. Early planting or delayed harvesting of farmland can cause significant fluctuations in the food resources available for Siberian cranes. Human harvesting operations also increase the vigilance behaviors of Siberian cranes, resulting in greater physiological stress and hindering their ability to replenish energy. Therefore, the reliance of Siberian cranes on dry farms for foraging poses substantial risks to their survival. To ensure the successful completion of spring and autumn migrations, the conservation efficiency of reserves should be improved, and suitable habitats should be incorporated into the protection system in a timely manner. National parks can solve this issue from a broader spatial perspective and through more effective management. Consequently, the concept and construction of Songnen Crane National Park are critical to and urgent for the protection of Siberian cranes.

Suitable habitat and habitat quality serve as two indispensable core criteria for identifying Siberian crane conservation gaps, and neither can be neglected. Moderately and highly suitable areas with land cover suitability values of 0.5–1 and high-quality habitat zones with habitat quality values ranging from 0.5 to 1 were extracted. Any overlapping patches located outside the boundaries of Momoge and Tumujji Nature Reserves were defined as key conservation gaps for Siberian cranes.

### 4.5. Conservation Implications

There is an ecological contradiction that highly suitable habitats for Siberian cranes are largely distributed across dry farmland with low overall habitat quality. Simply expanding protected boundaries cannot fully solve this conservation challenge. For wetland patches within reserves, priority should be given to hydrological regulation and wetland restoration to improve natural foraging conditions for Siberian cranes. For large areas of highly suitable farmland outside reserves, alternative management measures such as farmland ecological compensation, post-harvest residue retention, and reasonable farming schedule adjustment are more practical than strict enclosure protection. When planning the Songnen Crane National Park, multi-dimensional criteria including patch connectivity, threat intensity, land tenure and management feasibility should be integrated, rather than only relying on the proportion of suitable habitats outside protected areas.

### 4.6. Limitations of This Study

Several limitations should be acknowledged in this study. First, the outputs of the InVEST habitat quality model are sensitive to parameter settings including threat weights and maximum disturbance distances; parameter settings depend on field surveys and published regional literature, which inevitably contain subjective judgment. Second, for the MaxEnt model, although spatial thinning was applied to reduce sampling bias, field survey points may still be biased toward easily accessible roadside areas. Third, the spatial–temporal patterns of land cover change were analyzed, but quantitative attribution of driving forces (climate vs. human activity) was not performed. Future work could combine field food resource surveys and quantitative driving force decomposition to support more refined conservation decision making. Nevertheless, conservation priority planning for unprotected Siberian crane stopover sites should consider more spatial attributes beyond the joint output of habitat quality and species-specific suitability. Patch size, landscape connectivity, geographic distance from existing protected areas, long-term habitat stability, threat intensity, and on-site management feasibility are essential criteria for delineating new conservation zones.

## 5. Conclusions

The habitat quality and area of suitable habitats for Siberian cranes were examined in the Erlongtao River Basin over a 33-year period. The results indicate that land use types with relatively high-quality habitats, including swamps, shallow areas, forests, and water areas, were primarily distributed along the central axis of the northwestern and southeastern regions of the study area from 1990 to 2023. In contrast, land use types with poor-habitat quality, such as dry farm and unutilized land, were widely distributed throughout the northwestern part of the study area. The primary land use type for highly suitable habitat was dry farm located within and around the Momoge and Tumuji Nature Reserves and near areas connected to wetlands. With respect to high- and moderate-suitability habitat, 44% and 62% of the land cover classes were classified as “poor” in terms of land cover class quality, and 66% and 68% of these land cover classes were located outside the reserve. To ensure the long-term conservation of Siberian cranes and consider the role of habitat quality in ecological sustainability, restoring wetlands that contribute substantially to habitat quality is essential. Additionally, high-suitability areas for Siberian cranes should be reasonably protected. This study provides a reference for the conservation of endangered species and watershed ecological construction and offers scientific support and a theoretical foundation for the planning and development of Songnen Crane National Park. However, decision making for park construction should also consider land tenure, management capacity, and socioeconomic factors beyond habitat-related metrics. Several study limitations should be taken into account, including the parameter subjectivity of the InVEST model and potential sampling bias in species occurrence records. Future research could integrate field food resource surveys and quantitative driving force decomposition to deliver more targeted conservation strategies for Siberian cranes.

## Figures and Tables

**Table 1:** The maximum influence distance, weight and attenuation type of threat sources.

Threat	Maximum Influence Distance/km	Weight	Attenuation Type
Distance from the road	6	0.5	Linear
Construction land	5	1	Exponential
Unutilized land	2	0.4	Exponential

**Table 2:** Sensitivity index of land use type according to habitat threat factors.

Land Use	Habitat Adaptability	Sensitivity to Threat Factors		
		Distance from the Road	Unutilized Land	Construction Land
No data	0	0	0	0
Paddy fields	0.4	0.7	0.5	0.6
Dry farm	0.5	0.6	0.4	0.5
Forest land	0.6	0.7	0.5	0.6
Grasslands	0.4	0.7	0.7	0.4
Water area	0.7	0.6	0.4	0.8
Shallows	1	0.2	0.4	0.8
Construction	0	0	0	0
Unutilized land	0	0	0	0
Swamp	1	0.2	0.2	0.5

**Table 3:** Environmental factors.

	Driving Factors	Unit	Data Sources
Natural geographical factors	Soil type		http://www.resdc.cn
	NDVI		http://www.resdc.cn
	Distance from the road	meter	https://www.tianditu.gov.cn
	Distance from county and city	meter	https://www.tianditu.gov.cn
	Distance from water system	meter	https://www.tianditu.gov.cn
	Annual average temperature	°C	http://data.tpdc.ac.cn
	Elevation	meter	http://www.gscloud.cn
	Slope	degree	http://www.gscloud.cn
	Slope direction	degree	http://www.gscloud.cn
Socioeconomic factors	Population density	People per square kilometer	http://www.resdc.cn
	Per capita GDP	Ten thousand yuan	http://www.resdc.cn
	Land use		http://www.resdc.cn

**Table 4:** Area percentage proportions of various land use types in the study area (%).

Year	Paddy Fields	Dry Farm	Forestland	Grassland	Water Area	Shallows	Construction	Unutilized Land	Swamp
1990	0.06	28.82	6.98	41.01	2.97	0.07	1.93	10.88	7.27
1995	0.40	35.74	7.51	35.22	3.02	0.88	1.96	8.89	6.39
2000	0.59	36.17	7.16	35.40	2.30	0.07	1.95	8.53	7.84
2005	1.01	36.36	7.16	35.76	1.87	0.08	1.95	8.25	7.56
2010	2.35%	39.39%	7.29%	31.44%	1.78%	0.26%	1.75%	9.51%	6.23%
2015	2.53	39.57	7.29	31.22	2.01	0.26	1.76	9.34	6.03
2020	1.75	38.99	7.35	31.57	1.96	0.08	2.31	8.81	7.18
2023	0.77	36.93	7.04	34.80	5.83	0.11	2.31	5.71	6.51

**Table 5:** Area percentage proportions of different habitat quality levels in the study area (1990–2023) (%).

Year	1990	1995	2000	2005	2010	2015	2020	2023
Poor	15.99	13.43	13.66	13.18	14.26	13.97	14.15	7.92
Relatively poor	67.83	69.88	69.71	70.84	71.11	71.37	70.34	72.83
Good	8.47	9.57	8.72	8.34	8.14	8.37	8.32	12.71
Excellent	7.34	7.12	7.91	7.64	6.49	6.29	7.20	6.54

**Table 6:** Contributions and permutation importance (%).

Variable	Percent Contribution	Permutation Importance
Per capita GDP	28.8	22
Distance from the Class II Highway	11	10
Distance from the Class III Highway	10.3	7.6
Distance from water system	9.7	8.3
Land use	8.3	1.8
Annual average temperature	7.2	8.7
Distance from the Class IV Highway	6.2	6.1
Elevation	5.1	39.5
Distance from the Class I Highway	4.6	4.2
Soil type	2.4	2.9
Distance from county and city	2.1	3.1
NDVI	2	1
Slope direction	1.5	1.3
Slope	0.3	0.4
Population density	0.2	3.2

**Table 7:** Percentage of suitability levels for different land cover types (%).

	Land Cover							
	Paddy Fields	Dry Farm	Forestland	Grassland	Water Area	Construction	Unutilized Land	Swamp
Highly suitable habitat		47.97	5.77	4.81	0.07	2.46	36.83	2.08
Moderately suitable habitat	0.03	31.79	3.03	6.68	0.66	1.56	53.34	2.91
Low-suitability habitat	0.16	28.04	2.89	12.63	2.68	1.70	45.80	6.11
Unsuitable habitat	1.83	39.49	7.50	32.71	1.96	2.34	6.75	7.32

**Table 8:** Area of different suitable habitats inside and outside the protected area (km^2^).

	Tumuji Nature Reserve	Momoge Nature Reserve	Outside the Protected Area
Unsuitable habitat	974.20	182.92	6856.37
Low-suitability habitat	18.38	65.43	216.24
Moderately suitable habitat	5.89	31.42	79.06
Highly suitable habitat	1.70	3.43	9.86

## Data Availability

The original data presented in the study are openly available in the following public resources domain: http://www.resdc.cn; https://mulu.tianditu.gov.cn; http://data.tpdc.ac.cn; http://www.gscloud.cn/.

## Supplementary Materials

The following supporting information can be downloaded at: https://www.mdpi.com/article/10.3390/biology15191759/s1. Figure S1: The response curves of habitat suitability for Siberian cranes to various environmental factors; Table S1: The identification standards of the nine land-use categories.
