# Supplementary material for: Habitat Quality, Conservation Gaps, and Environmental Suitability of Siberian Cranes (Leucogeranus leucogeranus) in the Erlongtao River Basin, Songnen Plain
Source: Biology (Basel). 2026 Oct 2;15(19):1759. doi: 10.3390/biology15191759 (PMC13664573; doi:10.3390/biology15191759)
Supplement: Supplementary file 1 [file biology-15-01759-s001.zip › biology-4447187-supplementary.pdf]

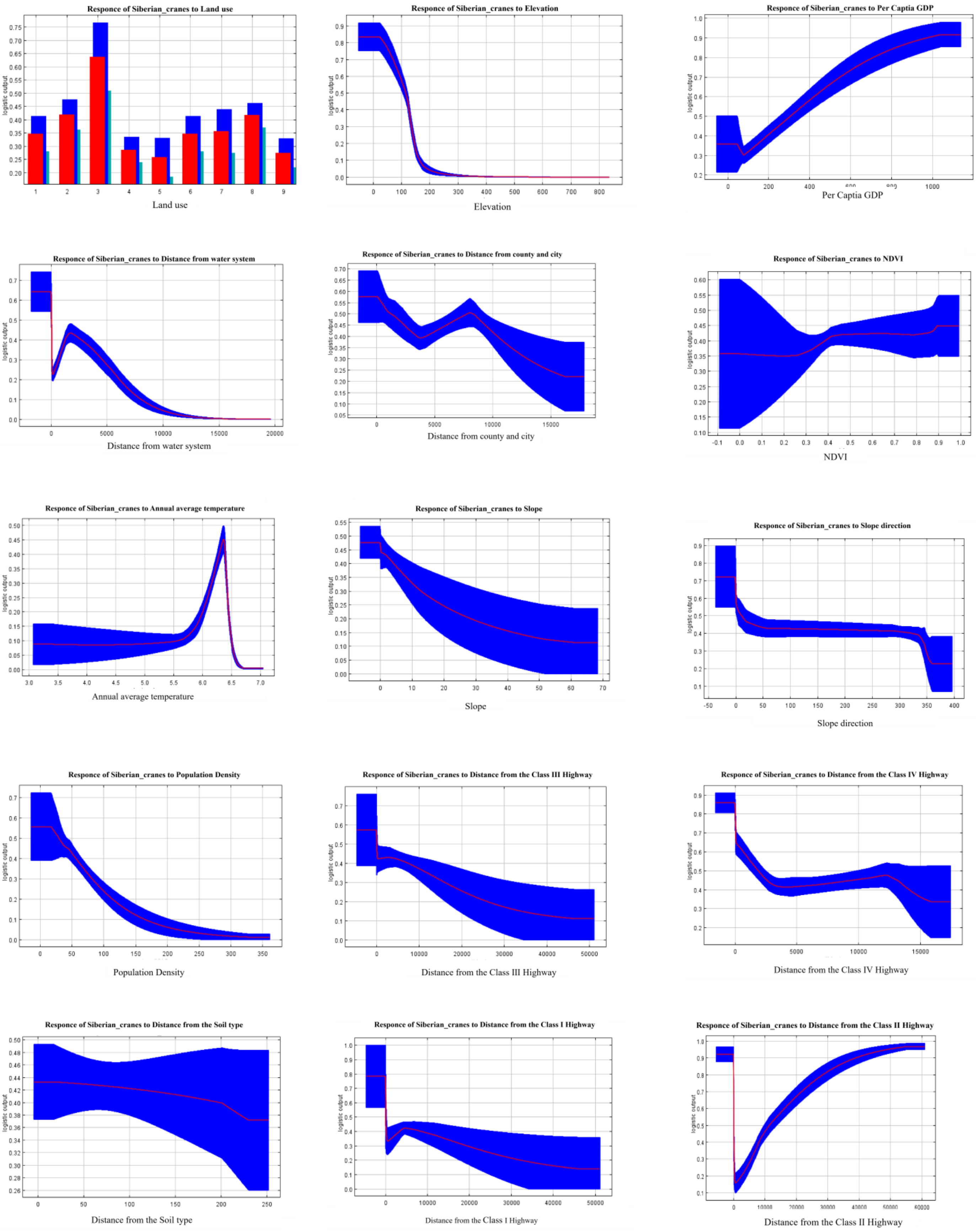

Figure S1. The response curves of habitat suitability for Siberian cranes to various environmental factors

Table S1. The identification standards of the nine land-use categories

Land-use category	Detailed identification standard
Paddy fields	Seasonal irrigated farmland equipped with water supply facilities for rice planting
Dry farm	Rain-fed cultivated land for corn, soybean and other dry crops
Forestland	Woodland, shrub land with vegetation coverage over 10%
Grasslands	Herbaceous vegetation areas including natural and saline grassland
Water areas	Permanent large water bodies such as lakes and rivers
Shallows	Seasonal mudflats and water-land ecotone beside open water
Construction	Urban/rural residential land, industrial land and roads
Unutilized land	Bare saline-alkali land without stable vegetation cover
Swamp	Long-term waterlogged areas dominated by acquatic wetland plants
